# The association between child maltreatment and emotional, cognitive, and physical health functioning in Vietnam

**DOI:** 10.1186/s12889-017-4258-z

**Published:** 2017-04-19

**Authors:** Nhu K. Tran, Sheila R. Van Berkel, Marinus H. van IJzendoorn, Lenneke R.A. Alink

**Affiliations:** Centre for Child and Family Studies, Leiden University, P.O. Box 9555, 2300 RB Leiden, The Netherlands

**Keywords:** Child maltreatment, Child abuse and neglect, Emotional problems, Physical health, Cognitive functioning, Vietnam

## Abstract

**Background:**

There is a paucity of research on correlates of child maltreatment in limited-resource countries with a relatively high tolerance of harsh discipline. This Vietnamese study aimed to investigate associations between different types of child maltreatment and child emotional, cognitive, and physical health functioning as well as moderation effects of gender and ethnicity.

**Methods:**

This cross-sectional study was conducted with 1851 randomly selected students aged 12–17 years. Both self-report and more objective measures (weight, height, study ranking, and a memory test) were used.

**Results:**

All types of child maltreatment were associated with emotional dysfunctioning. Life time and past year experiences of physical abuse and life time experiences of sexual abuse and neglect were related to poorer perceived physical health. The study did not find associations between any type of child maltreatment and overweight or underweight status. Regarding cognitive functioning, life time experience of sexual abuse and neglect were related to poorer working memory performance. Noticeably, emotional abuse was related to better academic performance, which might be an indication of “tiger parenting” practice in Vietnam, implying academic performance stimulation at the expense of emotional security. No significant moderation effects by gender and ethnicity were found.

**Conclusion:**

Even in a culture in which harsh discipline is normative, child maltreatment was related to negative aspects of child wellbeing including emotional, cognitive, and physical health functioning. Efficient and low-cost interventions on child maltreatment should be developed and conducted in Vietnam as well as other countries with similar contexts.

## Background

Child maltreatment has been associated with poor emotional, cognitive, and physical health functioning both during childhood and later in life [1–3]. Although child maltreatment exists in every culture and every society, research on child maltreatment focuses primarily on North American and European societies, and much less on Asian societies, especially those societies with limited resources [4]. In addition, most studies are unable to control for comorbidity of different types of child maltreatment, because they did not investigate all types of maltreatment [2, 3]. In the current study, we investigated the relation between five types of child maltreatment (sexual abuse, physical abuse, emotional abuse, witnessing parental conflict, and neglect) and child emotional, cognitive, and physical functioning while controlling for comorbidity among different types of child maltreatment in a representative sample in Vietnam.

The effects of different types of child maltreatment on emotional functioning have been studied frequently, mostly in Western high resource countries [2, 5]. A review of seven meta-analyses on the association between child sexual abuse and mental health showed that child sexual abuse increases the risk of depression, anxiety, post-traumatic stress disorder (PTSD), and impaired self-esteem in adulthood [6]. In addition, there is meta-analytic evidence for the association between physical abuse, emotional abuse, and neglect and a higher risk on depression and anxiety, and between physical abuse and PTSD and panic disorder [2]. In a meta-analysis on exposure to domestic violence, a relation was found between experiencing domestic violence, and emotional problems including depression, anxiety, PTSD, and internalizing disorders in general [5]. Moreover, there is empirical evidence that child maltreatment is linked to poorer interactions with peers [7].

In addition, there is some, although limited, evidence for the association between child maltreatment and cognitive development. Small-sized studies, mostly with clinical samples or samples referred by Child Protection Services found that emotional abuse [1], physical abuse, neglect, and witnessing domestic violence [8] were related to poorer spatial working memory. Furthermore, physical neglect was related to poorer recognition memory [1]. Physical abuse and sexual abuse were associated with impairments in verbal short-term memory [9] and working memory for positive emotions [10]. Controlling for comorbidity of other types of child maltreatment, sexual abuse was uniquely related to deficits in language and memory functioning [11]. However, a study conducted with a nationally representative sample from the United States found no effect of physical abuse or sexual abuse on memory performance, although the timing of the first experience of sexual abuse did account for differences in memory performance [12]. Compared with adults without sexual abuse experiences, adults with such an experience during early childhood performed better on a working memory test, while those who experienced sexual abuse only during adolescence performed worse [12]. Finally, child maltreatment was associated with lower academic performance as a proxy of cognitive functioning [13, 14].

Furthermore, child maltreatment seems to be related to physical health problems, although again, results are not always pointing in the same direction. A meta-analysis found that child maltreatment was associated with poorer adult health including neurological, musculoskeletal, respiratory, cardiovascular, gastrointestinal, and metabolic problems, more hospital visits, and more general health issues [3]. However, most of the participants of the studies in this meta-analysis were females, restricting generalizability of the findings to this gender. Another meta-analysis exploring the long term health consequences of child physical abuse, emotional abuse, and neglect showed that the associations with some health problems were weak and inconsistent and the number of studies on some of the other health outcomes was insufficient to be meta-analyzed [2]. In addition, there is meta-analytic evidence that physical, emotional, and sexual abuse are related to being overweight in adulthood [15], and studies have shown that neglect and emotional abuse are related to underweight status [16, 17] although this association is not always confirmed [18]. Most studies on the physical consequences of child maltreatment assess physical problems in adulthood. It is unclear whether physical health problems as a result of child maltreatment are already visible in childhood. Finally, similar to research on the emotional and cognitive consequences of maltreatment, most studies did not examine the effects of all types of child maltreatment [2, 3, 15]. Since about half of the maltreated children experience more than one type of child maltreatment [19, 20], it is essential to control for the other types of maltreatment in order to know how specific types of maltreatment relate to outcomes.

There is little research on the consequences of child maltreatment in Asian societies, especially those societies with limited resources. Vietnam is a middle-income country with a culture highly influenced by Confucianism. In Vietnam, the saying “Spare the rod, spoil the child” describes the spirit of parenting practices. Physical and emotional punishment are considered necessary to raise well-behaved children in Vietnam as well as many other countries in Asia [21]. Indeed, the prevalence of child maltreatment in Vietnam was found to be high, emotional abuse was most prevalent at 31.8%, followed by neglect (25.0%), physical abuse (19.1%), witnessing parental conflicts (15.3%) and sexual abuse (2.6%) [22]. These prevalence rates are consistent with similar countries in Asia and Pacific regions [23, 24], but compared directly to Western countries such as the Netherlands, most types of child maltreatment were much more common in Vietnam [22]. The question is whether different types of child maltreatment have similar effects in Vietnam compared to Western cultures. In a study with an international sample, perceived normativeness of harsh discipline lowered the negative effects of harsh discipline [25]. The differential effects of *maltreatment* based on perceived normativeness have not been investigated yet. It may be that maltreatment has less severe effects as well in cultures where physical and emotional punishment are more normative.

In Vietnam, three studies on the association of child maltreatment with emotional and physical health functioning have been conducted [20, 26, 27]. The results of these studies on emotional functioning were partially similar to those in Western cultures. When controlling for the other types of child maltreatment, emotional abuse was associated with depression, anxiety, and impaired self-esteem and neglect was related to depression [26] and anxiety [27]. Furthermore, sexual abuse was related to more depression, anxiety, and psychological distress while controlling for co-morbidity of physical abuse, emotional abuse, and neglect [27]. However, no significant associations were found for physical abuse and emotional abuse with emotional problems, when controlling for neglect and sexual abuse [27]. Regarding physical health outcomes of child maltreatment, a study in Vietnam showed that poorer perceived physical health was associated with emotional abuse for both boys and girls, and with physical abuse for girls only [26]. However, these studies only used self-reported outcomes and were conducted with non-random samples or samples only representative of a small area in Vietnam.

The effects of child maltreatment may not be the same for boys and girls. A meta-analysis found larger effect sizes for the association between child maltreatment and health issues in samples with only females compared to samples including both males and females [3]. However, meta-analyses on the association between child sexual abuse and adult mental health did not confirm significant moderation effects of gender [28, 29].

In addition to gender, ethnicity may play a role in the effects of child maltreatment on the wellbeing of victims. The findings of studies with American samples showed that the effects of child maltreatment were more severe in African-American children than in children from other racial groups on the likelihood of being arrested for violence in young adulthood [30], suffering from PTSD in adulthood [31], and developing internalizing and externalizing behavior in both adolescence and adulthood [32]. However, other studies found that the associations between child maltreatment and depression, heavy drinking, and violence were equivalent between Black and White adolescent and young adult men [33]. Differences in the effects of child maltreatment based on ethnicity have not yet been examined in Vietnam. The Kinh is the ethnic majority accounting for 84% of the population. We will test whether the association of maltreatment with different outcomes is different for Kinh versus non-Kinh populations.

In sum, the current study aims to (1) examine the association between sexual abuse, physical abuse, emotional abuse, witnessing parental conflict, and neglect and child emotional, cognitive, and physical health functioning in a representative sample in Vietnam using both self-report and more objective measures (weight, height, study ranking, digit span test); (2) test the moderation effects of gender and ethnicity in these associations.

## Methods

### Sample

The study was conducted in four provinces of Northern Vietnam, namely Hanoi, Nam Dinh, Ha Tinh, and Tuyen Quang (see also [22]). Hanoi, the capital of Vietnam, was selected because of its unique metropolitan characteristics. Regarding the other three provinces, one province was randomly selected from each of the three geographic areas of Northern Vietnam. In each province, we randomly selected two secondary schools and two high schools. Because the sample of Hanoi represents the largest metropolitan population, only schools in urban areas were selected in this province. In each of the other three provinces, one secondary school (for children aged 12–14 years) was selected from a list of schools in urban areas and the other secondary school from a list of schools in rural areas. In addition, for logistical reasons, we selected for each province the two high schools (for children aged 15–17 years) that were nearest to the secondary schools. We excluded schools for blind students, schools with fewer than 40 students per grade, and boarding schools where children live full time. We randomly selected one or two classes per grade of each participating school, depending on the number of students in a class.

Our sample thus consisted of a total of eight secondary schools and eight high schools. In total, 2360 students from 71 classes participated in the study. Students were excluded when unreliable answers were suspected based on outlying scores (z-scores greater than 3.29 [34]) on the Wildman Symptom Checklist, a scale consisting of bogus symptoms, such as “The buzzing in my ears keeps switching from the left to the right” [35, 36], (*n* = 31) or based on a specific pattern in their answers on the maltreatment questionnaire (e.g., all questions answered with the highest possible score; *n* = 23). In addition, students who were older than 17 (*n* = 331) or younger than 12 years (*n* = 2) or with missing data concerning age (*n* = 123) were excluded. The final sample consisted of 1851 students (47.3% boys, 57.6% secondary school students). The students were equally distributed among the four provinces. Most of the students were Kinh (81.7%), and 17.8% belonged to one of the ethnic minority groups (the other 0.5% had missing values for ethnicity). The mean age of the students was 14.2 years (*SD* = 1.4) and 89% of the students lived in two-parent families.

### Procedure

After the Provincial Department of Education (DOE) and the school boards approved the implementation of the study in the schools, informed consent was obtained from both the students and their parents. In Nam Dinh, Ha Tinh, and Tuyen Quang, passive informed consent was used. In Hanoi active consent was obtained which was a requirement of the DOE. One week prior to the study, students were provided an envelope for themselves and one for their parents. Each envelop had a letter with information about the study and a participation refusal form. In the active consent group, a letter of information with a participation form was provided. Students were asked to give one envelop to their parents. Only students who turned in both the participation refusal forms unsigned in the passive consent group and both participation forms signed in the active consent group could participate. Only 4.4% of the students and/or parents refused to participate through passive consent, while in the active consent procedure the refusal proportion was 18.1%.

The students filled out the questionnaire during class hours. Students who refused or students whose parents refused participation in the study filled out dummy questionnaires to avoid making these students a special group in the classroom. These questionnaires were not used in our analyses and destroyed after the study. The research proposal was approved by the Ethics Committee of the Institute of Education and Child Studies of Leiden University and the Ethics Committee of the Institute of Population, Health and Development of Vietnam.

### Measures

#### Child maltreatment

The child maltreatment questionnaire was based on the measure of the Prevalence Study on Maltreatment of children and youth in the Netherlands 2010 (NPM 2010; Euser et al. [19]). The 32-item NPM maltreatment measure was based on the Dating Violence Questionnaire [37] and the Parent-Child Conflict Tactics Scales (CTSPC; [38]. The scale consists of the following subscales: emotional abuse (1 item), physical abuse (8 items), witnessed parental conflict (7 items), sexual abuse (8 items), and neglect (8 items). The maltreatment items were embedded in a questionnaire with filler items, which concerned unpleasant and nasty incidents, nonviolent parental discipline (CTSPC, [38], and the social desirability items of the Dating Violence Questionnaire [37]. The Cronbach’s alpha of the whole maltreatment scale was .75. In addition, the Cronbach’s alphas of the child maltreatment subscales were also adequate (≥ .69).

#### Emotional dysfunctioning

Emotional dysfunctioning was computed as the mean of standardized scores of five (sub)scales measuring the following constructs (1) depression, (2) anxiety, (3) post-traumatic stress symptoms, (4) self-esteem, and (5) social and emotional functioning at school. The scale had a high internal consistency with a Cronbach’s alpha of .81. Depression and anxiety were measured with the 7-item depression subscale (Cronbach’s alpha = .82) and the 6-item anxiety subscale (Cronbach’s alpha = .81) of the Brief Symptom Inventory (BSI; [39]. The BSI assesses whether specific psychological symptoms occurred in the past week using a five-point Likert scale ranging from 1 (“*Not at all*”) to 5 (“*Extremely*”). Post-traumatic stress symptoms were measured with the 17- item Child PTSD Symptom Scale (CPSS) (Cronbach’s alpha = .85) [40]. 

Participants indicated how often several PTSD symptoms occurred in the last two weeks such as “*Having upsetting thoughts or images about the event that came into your head when you didn’t want them to*” on a four-point Likert scale ranging from 0 (“*Not at all or only at one time*”) to 3 (“*5 or more times a week/almost always*”). Self-esteem was assessed with the 10-item Rosenberg Self-Esteem Scale (RSES) consisting of items such as “*On the whole, I am satisfied with myself*” answered on a four-point Likert scale ranging from 1 (“*Strongly agree*”) to 4 (“*Strongly disagree*”) (Cronbach’s alpha = .73) [41]. Finally, social and emotional functioning at school was measured with the 6-item subscale of the Pediatric Quality of Life Inventory (PedsQL) measuring social interactions with peers (“*I have trouble getting along with other teens*”) and the ability to concentrate on tasks at school (“*It is hard for me to pay attention in class*”) in the past month on a four-point Likert scale ranging from 1 (“*Never*”) to 5 (“*Almost always*”) (Cronbach’s alpha = .71) [42].

#### Working memory

Working memory was measured with the verbal digit span task, which was administered in the classrooms [43]. The verbal digit span task includes a forward test and a backward test. In each test, an audiotape was played in which seven random number sequences were read aloud at the speed of one sequence per second. The first sequence had three digits and every subsequent sequence had one additional digit compared to the previous sequence, up to the length of nine digits. In the forward test, students were asked to write down each sequence in the exact order as broadcasted. In the backward test, participants had to write down each sequence in reversed order. In both tests, students could start writing *after* the sequence was finished. During the test, the researchers monitored students closely to identify students who did not follow the instructions (e.g., who wrote down the numbers during listening instead of after the whole sequence was presented, or who were observed to copy answers from fellow students). These students were excluded from analyses, resulting in 26.5% (*n* = 491) missing values on working memory.

#### Academic performance

Academic performance was measured using students’ study ranking of their previous school year, which they filled out on the questionnaire. In Vietnam students get a study ranking at the end of every school year, which is a rating of their average scores of courses with grades on a scale from 1 to 10 and their qualification of courses without grades including music, art and gymnastics. The classification ranges from 1 (“*Excellent”)* to 4 (*“Poor”*). Academic performance is considered excellent if (1) the average grade on all courses is higher than 8.0 (on a scale from 1 to 10), (2) either the average grade on literature or mathematics is higher than 8.0, (3) there are no courses with grades below 6.5, and (4) courses that do not assess performance with grades are completed successfully. The academic performance is good if (1) the average grade is between 6.5 and 8.0 (2) either the average grade on literature or mathematics is between than 6.5 and 8.0 (3) there are no courses with grades below 5.0, and (4) courses that do not assess performance with grades are completed successfully. The academic performance is average if (1) the average grade is between 5.0 and 6.5 (2) either the average grade on literature or mathematics is between 5.0 and 6.5, (3) there are no courses with grades below 3.5, and (4) courses that do not assess performance with grades are completed successfully. The academic performance is poor if (1) the average grade is between 3.5 and 5, and (2) there are no courses graded below the 2.0. [44]. There were no students with an academic performance lower than “poor” in this study. To facilitate interpretation, classifications were recoded, which resulted in higher scores indicating better academic performance, before statistical analyses.

#### Underweight and overweight status

Participants were asked to fill out their current weight and height. Body mass index (BMI) was calculated as weight in kilograms divided by height in meters squared [45]. This crude measure of BMI has been used as a standardized indicator for weight problems. The international cut-off points for underweight (thinness grade 1) and overweight by sex and age were used [46, 47]. There were 84 children with underweight and 181 children with overweight status in this sample.

#### Physical health

Physical health was measured with 25 items selected from the Child Health Questionnaire (CHQ-CF87; [48]. The items covered perceptions concerning the participants’ current general health condition (e.g., “In general, would you say your health is…”) and limitations in the participants’ performance of specific physical activities (e.g., walking for 15 min). The response options vary between items, therefore combined standardized mean scores were computed. The Cronbach’s alpha of the total scale was good, α = .78.

### Statistical procedures

As missing values were not missing completely at random (MCAR) and the percentage of missing values ranged from 0.3% (neglect) to 26.5% (working memory), we imputed the missing values using demographic variables (i.e. number of children in the family, living with both biological parents, war experiences father, and parental education and unemployment) in addition to all study variables. Multiple imputation was performed in SPSS 23 using all background, predictor, and outcome variables [49]. To deal with outliers, body mass index, physical health, and emotional wellbeing were winsorized before imputing missing data. One hundred data sets were imputed to allow for detection of small effect sizes even with a rather high percentage of missing values, as in case of working memory [50].

To explore the relation between child maltreatment and emotional, cognitive and physical health functioning of the children, hierarchical linear regression analyses with emotional dysfunction, working memory, academic performance, and physical health as outcome variables and logistic regression analyses were conducted with underweight and overweight status as outcome variables. To control for the effect of several background variables (age, gender, ethnicity, metropolitan level, and total items in the household, such as color television, motor bike as a measure for social economic status), these variables were added in the first step of the model. In the second step, child maltreatment variables were added. To test interaction effects between maltreatment and gender and ethnicity, similar regression analyses were run with the interaction variables of gender or ethnicity and child maltreatment in the third step (in separate models for gender and ethnicity). Separate models were tested for past year and lifetime maltreatment. Results are based on the pooled standardized regression coefficients of the 100 imputed data sets and the pooled explained variance that was computed by calculating the mean (pseudo) *R* ^2^ of the 100 imputed data sets. Because of the number of tests, we used a conservative significance level of alpha < .01.

## Results

### Associations between variables: Correlations

The correlations between background variables, child maltreatment variables, and outcome measures are presented in Table 1. Most types of maltreatment, both past year and life time experiences, were positively related to each other indicating that there was comorbidity among types of child maltreatment (Table 1). Only emotional abuse was not related to sexual abuse (both past year and life time), past year experience of emotional abuse was not associated with neglect, and life time experience of emotional abuse was not related to life time and past year experience of witnessing parental conflict. The life time experience of emotional abuse was negatively correlated with neglect.

**Table 1:** Correlations between predictors and outcome variables

	1	2	3	4	5	6	7	8	9	10	11	12	13	14	15	16	17	18	19
1. Age																			
2. Gender	-.02																		
3. Ethnic majority	-.01	-.04																	
4. Items household	-.07*	.09*	.43*																
5. Rurality	.07*	-.04	-.30*	-.44*															
6. Sexual abuse, P	-.05	.09*	-.10*	-.09*	.06														
7. Physical abuse, P	-.05	.08*	.05	.07	-.05	.21*													
8. Emotional abuse, P	.05	.03	.02	.08*	-.01	.07	.26*												
9. WPC, P	.04	-.01	-.02	-.05	.02	.19*	.17*	.11*											
10. Sexual abuse, L	-.02	.09*	-.10*	-.08	.06	.78*	.20*	.04	.15*										
11. Physical abuse, L	.04	.08*	.08*	.07	-.03	.14*	.60*	.16*	.14*	.16*									
12. Emotional abuse, L	.09*	-.05	.06	.06	-.00	-.00	.11*	.55*	.05	-.01	.17*								
13. WPC, L	.09*	-.01	.01	-.03	.04	.16*	.16*	.07*	.77*	.17*	.21*	.07							
14. Neglect, L	-.01	-.05	-.00	-.03	-.00	.10*	.13*	-.03	.08*	.12*	.12*	-.06*	.09*						
15. Emotional dysfunction	.17*	-.15*	.01	.01	.02	.07	.25*	.18*	.09*	.13*	.26*	.14*	.16*	.26*					
16. Working memory	.06	.00	.23*	.32*	-.18*	-.11*	.01	.08*	.03	-.12*	.04	.10*	.04	-.11*	-.04				
17. Academic performance	-.18*	-.12*	.27*	.36*	-.15*	-.06	.02	.04	-.02	-.06	.00	.09*	-.04	-.06	-.09*	.28*			
18. Underweight	-.02	.01	-.04	-.13*	.08*	.03	.02	-.02	.05	.02	.02	-.01	.05	.02	-.03	-.07*	-.08*		
19. Overweight	-.12*	.09*	.02	.10*	-.10*	.05	.05	-.02	.03	.03	07*	-.02	.01	.02	.00	.02	.03	-.06	
20. Physical health	-.08*	.19*	.13*	.17*	-.14*	-.08*	-.10*	-.06	-.06*	-.12*	-.12*	-.06*	-.11*	-.15*	-.15*	.14*	.14*	-.04	.03

*Note: * Gender (0 = girls, 1 = boys)

* *p* < .01

*WPC* witnessing parental conflict

*P* past year

*L* life time

Except for the relation between emotional dysfunction and working memory, better working memory, better physical health, higher academic performance and lower emotional dysfunction were related to each other. For underweight status, negative correlations with working memory, and academic performance were found.

More emotional dysfunction and poorer physical health were associated with most types of child maltreatment both past year and life time (Table 1), except emotional dysfunction, which was not related to past year sexual abuse, and physical health was not related to past year emotional abuse. Working memory was positively related to past year and life time emotional abuse, and negatively related to past year and life time sexual abuse and neglect. Academic performance was positively associated with life time emotional abuse. Overweight was positively related to life time physical abuse.

### Associations between maltreatment and emotional, cognitive, and physical health functioning

The findings of the regression analysis predicting emotional dysfunctioning showed that all types of child maltreatment during life time were associated with more emotional dysfunctioning (Table 2). With respect to past year child maltreatment experience, only physical abuse and emotional abuse were positively associated with emotional dysfunctioning.

**Table 2:** Results of the regression models predicting emotional dysfunction

		Past year maltreatment		Life time maltreatment	
		*R* ^*2*^ *=* .13		*R* ^*2*^ *=* .19	
Model		Beta	*p*	Beta	*p*
1	Age	.17*	.00	.15*	.00
	Gender	-.17*	.00	-.16*	.00
	Ethnic majority	-.01	.60	-.03	.30
	Total items in the household	.02	.38	.04	.12
	Rurality	.02	.51	.01	.66
2	Physical abuse	.24*	.00	.19*	.00
	Sexual abuse	.04	.19	.09*	.00
	Emotional abuse	.11*	.00	.10*	.00
	Witnessing parental conflict	.02	.28	.07*	.00
	Neglect ª			.22*	.00

*Note*. Betas and *R* ^*2*^ in model 1 and model 2 are derived from model 2, *R* ^*2*^ is the mean of the *R* ^*2*^ for the imputed data sets

Gender (0 = girls, 1 = boys)

* *p* < .01

ª Neglect only covered life time experiences

For cognitive functioning, the regression analysis predicting working memory showed negative main effects of life time experience of sexual abuse and neglect (Table 3). The experience of sexual abuse and neglect were associated with poorer working memory. Furthermore, the regression analyses predicting academic performance showed that life time emotional abuse was associated with better academic performance of the participants (Table 4).

**Table 3:** Results of the regression models predicting working memory

		Past year maltreatment		Life time maltreatment	
		*R* ^*2*^ *=* .10		*R* ^*2*^ *=* .12	
Model		Beta	*p*	Beta	*p*
1	Age	.07*	.005	.07*	.007
	Gender	-.01	.63	-.01	.56
	Ethnic majority	.10*	.00	.09*	.00
	Total items in the household	.25*	.00	.25*	.00
	Rurality	-.04	.13	-.05	.09
2	Physical abuse	-.01	.66	.02	.60
	Sexual abuse	-.08	.02	-.08*	.005
	Emotional abuse	.06	.04	.06	.03
	Witnessed parental conflict	.05	.06	.05	.04
	Neglect ^a^			-.10*	.00

*Note*. Betas and *R* ^*2*^ in model 1 and model 2 are derived from model 2, *R* ^*2*^ is the mean of the *R* ^*2*^ for the imputed data sets

Gender (0 = girls, 1 = boys)

* *p* < .01

^a^ Neglect only covered life time experiences

**Table 4:** Results of the regression models predicting academic performance

		Past year maltreatment		Life time maltreatment	
		*R* ^*2*^ *=* .18		*R* ^*2*^ *=* .19	
Model		Beta	*p*	Beta	*p*
1	Age	-.16*	.00	-.16*	.00
	Gender	-.14*	.00	-.14*	.00
	Ethnic majority	.13*	.00	.14*	.00
	Total items in the household	.31*	.00	.31*	.00
	Rurality	.03	.23	.03	.26
2	Physical abuse	-.01	.72	-.01	.61
	Sexual abuse	-.02	.40	-.00	.95
	Emotional abuse	.03	.21	.07*	.00
	Witnessed parental conflict	-.01	.78	-.02	.45
	Neglect ^a^			-.05	.02

*Note*. Betas and *R* ^*2*^ in model 1 and model 2 are derived from model 2, *R* ^*2*^ is the mean of the *R* ^*2*^ for the imputed data sets

Gender (0 = girls, 1 = boys)

* *p* < .01

^a^ Neglect only covered life time experiences

The two logistic regression analyses predicting weight problems found no significant associations between any type of child maltreatment and weight problems (Tables 5). Finally, past year and life time physical abuse experience and life time sexual abuse and neglect were associated with lower quality of physical health (Table 6).

**Table 5:** Results of the logistic regression predicting underweight and overweight status

		Past year maltreatment				Life time maltreatment			
		*Nagelkerke R* ^*2*^ = .10				*Nagelkerke R* ^*2*^ = .10			
		*B*	*SE*	*p*	Odds ratio	*B*	*SE*	*p*	Odds ratio
Underweight status									
1	Age	-.13	.13	.31	0.88	-.15	.13	.25	0.86
	Gender	.34	.13	.01	1.40	.34	.13	.01	1.41
	Ethnic majority	.20	.13	.11	1.22	.19	.13	.13	1.21
	Total items in the household	-.84*	.19	.00	0.43	-.85*	.19	.00	0.43
	Rurality	.14	.13	.30	1.15	.13	.13	.34	1.14
2	Physical abuse	.09	.14	.49	1.10	.06	.13	.64	1.06
	Sexual abuse	-.05	.17	.75	0.95	-.07	.15	.63	0.93
	Emotional abuse	-.12	.14	.41	0.89	-.01	.14	.95	0.99
	Witnessed parental conflict	.15	.12	.20	1.16	.19	.12	.12	1.21
	Neglectª					.07	.12	.57	1.07
Overweight status									
1	Age	-.47*	.11	.00	0.62	-.50*	.11	.00	0.61
	Gender	.33*	.11	.00	1.39	.32*	.11	.00	1.38
	Ethnic majority	-.13	.13	.33	0.88	-.14	.13	.26	0.87
	Total items in the household	.24	.12	.04	1.27	.22	.12	.06	1.25
	Rurality	-.41*	.14	.00	0.66	-.41*	.14	.00	0.66
2	Physical abuse	.10	.10	.34	1.10	.22	.11	.04	1.25
	Sexual abuse	.11	.11	.33	1.11	.05	.11	.65	1.05
	Emotional abuse	-.16	.11	.16	0.85	-.08	.10	.46	0.93
	Witnessed parental conflict	.11	.10	.28	1.12	.07	.11	.52	1.07
	Neglect^a^					.05	.10	.60	1.05

*Note*. Statistics in model 1 and model 2 are derived from model 2, *Nagelkerke R* ^*2*^ is the mean of the *Nagelkerke R* ^*2*^ for the imputed data sets

Gender (0 = girls, 1 = boys)

* *p* < .01

*B* is comparable with Beta as all the variables were standardized

^a^ Neglect only covered life time experiences

**Table 6:** Results of the regression models predicting physical health

		Past year maltreatment		Life time maltreatment	
		*R* ^*2*^ *=* .09		*R* ^*2*^ *=* .12	
Model		Beta	*p*	Beta	*p*
1	Age	-.07*	.00	-.06	.01
	Gender	.19*	.00	.19*	.00
	Ethnic majority	.07*	.005	.08*	.00
	Total items in the household	.10*	.00	.09*	.00
	Rurality	-.07*	.009	-.06	.01
2	Physical abuse	-.11*	.00	-.11*	.00
	Sexual abuse	-.05	.09	-.08*	.00
	Emotional abuse	-.03	.14	-.04	.08
	Witnessed parental conflict	-.02	.33	-.05	.04
	Neglect ^a^			-.11*	.00

*Note*. Betas and *R* ^*2*^ in model 1 and model 2 are derived from model 2, *R* ^*2*^ is the mean of the *R* ^*2*^ for the imputed data sets

Gender (0 = girls, 1 = boys)

* *p* < .01

^a^Neglect only covered life time experiences

### Moderating effects of gender and ethnicity

The examination of the moderations by gender and ethnicity showed that there were no significant moderation effects on the association between child maltreatment experiences and emotional, cognitive, and physical health functioning.

## Discussion

We showed that child maltreatment was related to different aspects of child wellbeing including emotional, cognitive, and physical health functioning. More specifically, controlling for all other types of maltreatment, we found that life time sexual abuse and neglect were related to emotional dysfuctioning, poorer working memory, and physical health problems. Past year and life time physical abuse was related to emotional dysfunctioning and physical health problems. Past year and life time emotional abuse was associated with emotional dysfuctioning. Life time prevalence of witnessing parental conflict was linked to emotional dysfunctioning. Contrary to our hypotheses, we found that life time emotional abuse was positively associated with academic performance and no associations were found for weight status.

All types of child maltreatment were associated with emotional dysfunctioning. This finding is consistent with previous research in mostly Western countries [2, 5, 6, 51]. Regarding cognitive functioning, sexual abuse and neglect were associated with poorer working memory performance. This is in line with several other studies on memory functioning [1, 8, 11]. Physical health was related to physical abuse, sexual abuse, and neglect. This finding extends previous findings on the effects of child maltreatment on physical health in adulthood [2, 3] by showing that also during childhood, physical abuse, sexual abuse, and neglect were associated with poorer perceived physical health while controlling for the effects of other types of child maltreatment.

Comparing our results with previous studies on the effects of child maltreatment in Vietnam reveals both similarities and differences. Earlier research in Vietnam found negative associations of some specific psychological conditions, for example depression and anxiety, with emotional abuse [26], sexual abuse [27], and neglect [26, 27]. The current study shows that - in addition to emotional abuse, sexual abuse, and neglect - physical abuse and witnessing parental conflict were also related to emotional dysfunctioning while controlling for the co-morbidity of other types of child maltreatment. In addition, previous research with Vietnamese students of similar age as our sample found that physical health problems were linked to physical abuse (for girls) and emotional abuse [26]. Our results extend these findings in that we also found associations with sexual abuse and neglect. Our measure of physical health was a general one whereas Nguyen (2006) measured specific physical health problems, which may explain differences in effects. Contrary to this earlier study however, we did not find an association with emotional abuse which could possibly be explained by the fact that in the current study emotional abuse was measured with a single item, which may not be representative of the full range of emotionally abusive behaviors. In addition, our study is the first to investigate and find associations between child maltreatment and cognition in Vietnam. Taking these and our other findings together, we showed that the consequences of child maltreatment in Vietnam seem to be more extensive than was thought based on previous research. Considering the high prevalence of all types of child maltreatment in Vietnam, with prevalence rates up to 39.0% for physical abuse and 59.6% for emotional abuse [22], child maltreatment is a very serious threat to the general health of the Vietnamese population.

One of the central mechanisms of the association between child maltreatment and emotional, cognitive, and physical health outcomes may be alterations in the stress system and brain areas regulating the stress system. There is a wide range of evidence that the chronic stress of child maltreatment can induce long term impairments of the hypothalamic-pituitary-adrenal axis (HPA axis), a core part of the human stress system [7, 52]. In turn, changes in the functioning of the HPA-axis are related to mental health problems such as anxiety and depression [53]. Moreover, abnormal cortisol levels as a result of an impaired stress response system were also associated with lower cognitive functioning [54]. More specifically, meta-analytic results showed that child maltreatment was associated with reduced hippocampal volume, a brain area that plays an important role in cognition, primarily memory, and controls the regulation of the HPA axis [55]. In addition, impaired functioning of the HPA axis was found to alter metabolic processes, enhance the susceptibility to inflammation, and impair counter-regulatory control of immune responses [56], all putting individuals at risk for health problems. Another possible mechanism through which child maltreatment could influence emotional, cognitive, and physical health functioning is through an increase in exhibiting risky behaviors. Child maltreatment experiences were found to increase the practice of risky behaviors, such as substance abuse, smoking, physical inactivity, multiple sex partners, which in turn can impair emotional, cognitive, and physical health functioning [57].

Regarding physical and emotional abuse, not only life time experience, but also past year experience was related to emotional dysfunction, whereas for the other types of maltreatment only life time experiences were related to the outcomes. This finding suggests that past as well as recent experiences of physical and emotional abuse in adolescence can have devastating effects, whereas for the other types of maltreatment, the occurrence earlier in development may be more relevant. A reason that we did not find an association between past year sexual abuse and emotional dysfunction could be the low prevalence of sexual abuse in the past year which may not have allowed to detect significant association.

Among all types of child maltreatment, life time physical abuse and neglect were related to the largest number of dysfunctional child outcomes. While physical abuse has been studied frequently, research on neglect is often “neglected” [58]. Our finding on the effects of neglect is consistent with the results of Spinhoven et al. (2010) [59] that neglect has as much or even more detrimental effects than other types of child maltreatment, even after controlling for co-morbidity. Noticeably, total items in the household as a proxy for poverty was also related to four out of six outcome measures: physical health, underweight status, working memory performance, and academic performance. This finding may add to the findings on neglect and provides empirical evidence for the negative effect of poverty on cognitive functioning and physical health during childhood.

In contrast to our hypotheses, emotional abuse was related to better academic performance. Samples of previous studies reporting worse academic performance as a result of emotional abuse, all originated from Western and Middle Eastern countries and none originated from an East Asian country [13, 14]. In East Asian countries, such as Vietnam, the so called “tiger parenting” practice is more common than in countries in other regions [60, 61]. “Tiger parenting” was first introduced by Chua (2011) who described it as a harsh parenting style that focuses on making children reach high academic achievements and compliance with family obligations [62]. This parenting style is in line with the “achievement/adjustment paradox” observed in Asian American children, who both reach higher academic achievement and have more psychological problems [63], something that was also confirmed by the positive association between emotional abuse and emotional dysfunctioning found in this study. “Tiger parenting” has rarely been studied with Vietnamese parents; a qualitative study with a few Vietnamese-Australian mothers did find that Vietnamese parents had an authoritarian parenting style and expected high education achievements of their children [64]. Although there are no studies on “tiger parenting” conducted in Vietnam and our study did not measure parenting style, the association between emotional abuse and better academic performance on the one hand and worse emotional functioning on the other hand might be an indication of “tiger parenting” practice. As mentioned before, only one item was used to assess emotional abuse and therefore this association needs to be further investigated in future research.

Regarding overweight and underweight status, the current study did not find associations with any type of child maltreatment. Although previous findings on the effects of child maltreatment on underweight status are inconsistent, there is evidence that child maltreatment is related to more overweight in children referred to Child Protective Services [65] and there is meta-analytic evidence concerning this relation in adult samples [15]. The current study was conducted with a general population sample of school-aged children, and the effects of child maltreatment on overweight could be less explicit in population-based samples compared to children referred by Child Protective Services.

No significant results were found for the moderation effects of gender and ethnicity. A reason that we did not find moderation by ethnicity could be the differences between minor and major ethnicities in Vietnam mainly reflect differences in economic status, so after controlling for the effects of economic status and other background information differences, the effects of child maltreatment across ethnicities were similar. These findings imply that the effects of child maltreatment during childhood were equivalent across different genders and ethnicities after controlling for socioeconomic inequalities.

Some limitations of this study should be mentioned. First, the cross-sectional design of this study does not allow the examination of causality of child maltreatment on emotional functioning, cognitive functioning and physical health status. However, research with animal models and a longitudinal twin-study on child development provide strong evidence of causal effects of child maltreatment on different outcomes [66, 67]. Second, the different informed consent procedure in Hanoi compared with other provinces resulted in a lower participation rate for this region. The use of different consent procedures in one sample could affect the validity of the results of the study. Third, in terms of measurement, the emotional abuse scale only consisted of one item which limits the reliability and validity of this scale. Moreover, overweight and underweight status were identified through height and weight reported by the participants. It might be difficult for children to remember their precise height and weight, thus recall bias may affect the accuracy of weight problems in this study. Another limitation is the fact that a large percentage of working memory test results were invalid or missing (26.5%). Although we used a reliable multiple imputation process, the large number of missing values may still have introduced bias in the data analysis relating to working memory.

## Conclusion

This study contributes to the scarce empirical research on the effects of child maltreatment in limited resource contexts such as Vietnam. We showed that the consequences of maltreatment may be more widespread than previously thought. There were several associations of child maltreatment with different domains of child wellbeing including emotional, cognitive, and physical health functioning, even when controlling for co-morbidity. Among various types of child maltreatment, life time experience of sexual abuse, and neglect were related to the largest number of problems in functioning. Given these results and the shortage of studies specifically on neglect, more research on the effects of neglect, controlling for other types of child maltreatment, should be conducted. In addition, the positive association between emotional abuse and academic performance should be explored further.

In conclusion, considering the widespread consequences and the high prevalence rates of child maltreatment in Vietnam [22], we recommend that effective and low-cost interventions are designed and implemented to prevent or relieve the short- and long-term consequences of maltreatment.

## Abbreviations

BMI: Body mass index
BSI: Brief symptom inventory
CHQ: CF87 child health questionnaire
CPSS: Child PTSD symptom scale
CTSPC: Parent-child conflict tactics scales
HPA: Axis hypothalamic-pituitary-adrenal axis
MCAR: Missing completely at random
NPM: Netherlands’ prevalence study on maltreatment of children and youth
PedsQL: Pediatric quality of life inventory
PTSD: Post traumatic stress disorder
RSES: Rosenberg self-esteem scale
SD: Standard deviation
WPC: Witnessing parental conflict
